# Markers of Natural Killer Cell Exhaustion in HIV/HCV Coinfection and Their Dynamics After HCV Clearance Mediated by Direct-Acting Antivirals

**DOI:** 10.1093/ofid/ofad591

**Published:** 2023-11-22

**Authors:** Ariel Osegueda, Maria Laura Polo, Lucia Baquero, Alejandra Urioste, Yanina Ghiglione, Silvia Paz, Gabriela Poblete, Virginia Gonzalez Polo, Gabriela Turk, Maria Florencia Quiroga, Natalia Laufer

**Affiliations:** CONICET-Universidad de Buenos Aires, Instituto de Investigaciones Biomédicas en Retrovirus y SIDA (INBIRS), Buenos Aires, Argentina; Universidad de Buenos Aires, Facultad de Medicina. Buenos Aires, Argentina; CONICET-Universidad de Buenos Aires, Instituto de Investigaciones Biomédicas en Retrovirus y SIDA (INBIRS), Buenos Aires, Argentina; Universidad de Buenos Aires, Facultad de Medicina. Buenos Aires, Argentina; CONICET-Universidad de Buenos Aires, Instituto de Investigaciones Biomédicas en Retrovirus y SIDA (INBIRS), Buenos Aires, Argentina; Universidad de Buenos Aires, Facultad de Medicina, Departamento de Microbiología, Parasitología e Inmunología, Buenos Aires, Argentina; CONICET-Universidad de Buenos Aires, Instituto de Investigaciones Biomédicas en Retrovirus y SIDA (INBIRS), Buenos Aires, Argentina; Universidad de Buenos Aires, Facultad de Medicina. Buenos Aires, Argentina; CONICET-Universidad de Buenos Aires, Instituto de Investigaciones Biomédicas en Retrovirus y SIDA (INBIRS), Buenos Aires, Argentina; Universidad de Buenos Aires, Facultad de Medicina. Buenos Aires, Argentina; Hospital Francisco Javier Muñiz, Buenos Aires, Argentina; Hospital Francisco Javier Muñiz, Buenos Aires, Argentina; CONICET-Universidad de Buenos Aires, Instituto de Investigaciones Biomédicas en Retrovirus y SIDA (INBIRS), Buenos Aires, Argentina; Universidad de Buenos Aires, Facultad de Medicina. Buenos Aires, Argentina; CONICET-Universidad de Buenos Aires, Instituto de Investigaciones Biomédicas en Retrovirus y SIDA (INBIRS), Buenos Aires, Argentina; Universidad de Buenos Aires, Facultad de Medicina, Departamento de Microbiología, Parasitología e Inmunología, Buenos Aires, Argentina; CONICET-Universidad de Buenos Aires, Instituto de Investigaciones Biomédicas en Retrovirus y SIDA (INBIRS), Buenos Aires, Argentina; Universidad de Buenos Aires, Facultad de Medicina, Departamento de Microbiología, Parasitología e Inmunología, Buenos Aires, Argentina; CONICET-Universidad de Buenos Aires, Instituto de Investigaciones Biomédicas en Retrovirus y SIDA (INBIRS), Buenos Aires, Argentina; Universidad de Buenos Aires, Facultad de Medicina, Departamento de Microbiología, Parasitología e Inmunología, Buenos Aires, Argentina

**Keywords:** direct-acting antivirals, HIV/HCV coinfection, immunology, liver fibrosis, NK cell exhaustion

## Abstract

**Background:**

Liver fibrosis is a leading cause of morbimortality in people with HIV/hepatitis C virus (HCV). Natural killer (NK) cells are linked with amelioration of liver fibrosis; however, NK cells from individuals coinfected with HIV/HCV with cirrhosis display impaired functionality and high PD-1 expression. Here, we aimed to study PD-1, TIGIT, and Tim3 as potential exhaustion markers in NK cells from persons coinfected with HIV/HCV with mild and advanced liver fibrosis. We also evaluated the role of PD-1 expression on NK cells after HCV clearance by direct-acting antivirals (DAAs).

**Methods:**

Peripheral blood mononuclear cells were isolated from individuals coinfected with HIV/HCV (N = 54; METAVIR F0/F1, n = 27; F4, evaluated by transient elastography, n = 27). In 26 participants, samples were collected before, at the end of, and 12 months after successful DAA treatment. The frequency, immunophenotype (PD-1, TIGIT, and Tim3 expression), and degranulation capacity (CD107a assay) of NK cells were determined by flow cytometry.

**Results:**

Unlike PD-1, Tim3 and TIGIT were comparably expressed between persons with mild and advanced fibrosis. Degranulation capacity was diminished in NK/TIGIT^+^ cells in both fibrosis stages, while NK/PD-1^+^ cells showed a lower CD107a expression in cirrhotic cases. Twelve months after DAA treatment, those with advanced fibrosis showed an improved NK cell frequency and reduced NK/PD-1^+^ cell frequency but no changes in CD107a expression. In individuals with mild fibrosis, neither PD-1 nor NK cell frequency was modified, although the percentage of NK/CD107a^+^ cells was improved at 12 months posttreatment.

**Conclusions:**

Although DAA improved exhaustion and frequency of NK cells in cirrhotic cases, functionality was reverted only in mild liver fibrosis, remarking the importance of an early DAA treatment.

## Teaser

- PD-1 and TIGIT are associated with impaired natural killer (NK) cell functionality in individuals coinfected with HIV/HCV.
- NK cell degranulation capacity improved in persons with mild fibrosis after direct-acting antiviral treatment.
- In cases of cirrhosis, although frequency of NK cells was restored, loss of functionality was not.

Nearly 71 million people worldwide are chronically infected with hepatitis C virus (HCV) [1]. Estimates indicate that 25% to 30% of those will develop cirrhosis within 20 years of infection [2]. Among other factors, HIV coinfection has been demonstrated to significantly accelerate fibrosis progression and development of end-stage liver disease [3, 4], even in the presence of antiretroviral therapy [3]. With a global assessment of 2.3 million people coinfected with HIV/HCV [5], monitoring hepatic disease and preventing liver damage constitute essential recommendations for the clinical management of this population [6].

Direct-acting antivirals (DAAs) represent a major advancement in the treatment of HCV infection, since HCV clearance can be achieved in >90% of the cases, with minimal side effects [7]. However, the impact of DAA treatment on hepatic fibrosis still needs to be evaluated in larger populations and broader follow-ups [8]. Currently, studies show that DAA treatment is associated with the resolution of hepatic inflammation and improvement of fibrosis, especially among persons with mild to moderate levels of liver damage. Nevertheless, a great proportion of those with baseline advanced liver disease stay within cirrhotic scores [8]. Unraveling the mechanisms that regulate liver fibrosis is vital since nearly half a million people die annually from decompensated cirrhosis related to chronic HCV infection [1]. Moreover, end-stage liver disease constitutes a major cause of death among persons coinfected with HIV/HCV [9].

Natural killer (NK) cells play an important role in inhibiting hepatic fibrosis by killing early activated or senescence-activated hepatic stellate cells [10]. It has been shown that peripheral NK cell frequency is significantly decreased in HCV monoinfection and HIV/HCV coinfection [11, 12]. The functionality of these cells has also been reported to be severely compromised in both groups [11, 13, 14]. Previously, we demonstrated that persons coinfected with HIV/HCV with advanced fibrosis are characterized by having low peripheral NK cell frequencies as compared with healthy volunteers and those with minimal fibrosis.

Furthermore, NK cell degranulation and cytokine secretion were significantly diminished in samples from patients with higher levels of fibrosis. When PD-1 expression was assessed on the NK cell compartment, PD-1 expression was significantly upregulated in cirrhotic cases, despite presenting similar times of known HIV and HCV infection, time of antiretroviral therapy, HCV viral load, and HCV genotype to those observed in cases of mild hepatic disease [12, 15].

The PD-1 molecule has been extensively studied in T cells, B cells, and dendritic cells [16]; however, less is known about PD-1 expression and NK cell functionality. Several studies have shown that PD-1 is highly expressed on peripheral and tumor-infiltrating NK cells in patients experiencing different malignancies and that PD-1 axis blocking significantly enhances the cytokine production, degranulation, and viability of these cells [17]. PD-1 expression on NK cells was also linked to chronic HCV infection [18], although the relationship between progression of liver fibrosis and the PD-1 axis in NK cells has not been completely addressed. Additional surface proteins have been evaluated as potential NK cell immune checkpoints, including CD96, LAG3, T-cell immunoglobulin, mucin domain–containing 3 (Tim3), and T-cell immunoreceptor with Ig and ITIM domains (TIGIT) [19–21]. Of those, Tim-3 and TIGIT have been studied in viral hepatitis, and it has been suggested that expression of these markers is linked to exhausted phenotypic characteristics [22].

The aim of this work was to characterize PD-1, TIGIT, and Tim3 as potential biomarkers for NK cell exhaustion among persons coinfected with HIV/HCV with different degrees of liver fibrosis. In this regard, we evaluated the association between NK cell exhaustion and progression of liver damage. Additionally, we explored how baseline hepatic fibrosis levels affect PD-1 expression and NK cell dynamics following HCV eradication by DAA. By studying peripheral blood mononuclear cells (PBMCs) from individuals coinfected with HIV/HCV with mild and advanced liver fibrosis, we showed that PD-1 is a selective marker of NK cell exhaustion and is significantly linked to advanced fibrosis stages. Last, although NK cell frequency is mildly improved, HCV clearance does not completely restore NK cell functionality in cases of HIV/HCV coinfection and advanced liver fibrosis.

## METHODS

### Study Cohort

This study included individuals who were coinfected with HIV/HCV (n = 54; 27 with METAVIR F0/F1 and 27 with F4), HIV seropositive (HIV^+^, n = 6), HCV seropositive (HCV^+^, n = 9), and HIV/HCV seronegative (healthy controls, n = 5). Written informed consent was obtained, and 60 mL of peripheral blood was drawn. The study was conducted in accordance with the Declaration of Helsinki and was approved by the Bioethics Committee of Fundación Huésped. Persons coinfected with HIV/HCV were allocated to 2 groups based on their levels of fibrosis according to transient hepatic elastography (FibroScan and SuperSonic Imagine's Aixplorer). Those with a result ≤7.1 kPa were classified as compatible with a METAVIR score of F0/F1 (absent or minimal fibrosis) and those with ≥12.5 kPa as compatible with F4 (cirrhosis) [23]. Participants enrolled in this study were not acutely or chronically HBV infected (determined by serology) and denied current use of recreational drugs and 14 units per week of alcohol intake on a regular basis. From the 54 individuals who were coinfected, 26 blood samples (14 with F0/F1 and 12 with F4) were collected at 3 times: baseline (prior to DAA treatment), end of treatment (EOT), and 12 months posttreatment (12MPT).

### Cell Isolation and Culture

PBMCs were obtained from whole blood by Ficoll-Hypaque centrifugation (GE Healthcare) and cryopreserved at −80 °C for up to 4 months. Cells were cultured in complete RPMI-1640 medium (cRPMI) containing 10% fetal bovine serum, 2mM L-glutamine, 100 IU/mL of penicillin, and 100 μg/mL of streptomycin (all reagents, Gibco SRL). Three days before the experiments, the chronic myelogenous leukemia K562 cell line was thawed and grown at 37 °C and 5% CO_2_ in cRPMI.

### CD107a Assay

For degranulation assays, the K562 cell line was used as a stimulus and a sensible target for NK cells. PBMCs were thawed and cultured for 2 hours in cRPMI, at 37 °C with 5% CO_2_. Next, 1 million viable PBMCs were coincubated for 5 hours with 10^5^ K562 cells, in the presence of anti-CD107a-FITC mAb, brefeldin, and monensin (4 μL, 10 μg/mL, and 0.7 μg/mL, respectively; BD Biosciences). To assess basal levels of degranulation, PBMCs were incubated in the absence of K562 cells.

### Multicolor Flow Cytometry

Cells were immunophenotyped by flow cytometry on a FACS Canto Flow Cytometer (BD Biosciences). For antibodies and gating strategies, see Supplementary Table 1 and Supplementary Figure 1. Flow cytometry data was analyzed with FlowJo software version 10 (BD Biosciences). Phenotype and functionality assays were performed according to cell availability.

### Data Analysis

Statistical analysis was performed with Prism version 8 (GraphPad). Data normality was assessed with the Shapiro-Wilk test and subsequently analyzed by nonparametric methods. All tests were considered significant when *P* < .05.

## RESULTS

To evaluate biomarkers for NK cell exhaustion and its relationship with liver fibrosis, blood samples were obtained from persons coinfected with HIV/HCV with mild and advanced hepatic fibrosis, as assessed by transient elastography and METAVIR staging. Only participants with extreme F0/F1 and F4 levels were included in this study. This strategy was used to minimize METAVIR misclassification by transient hepatic elastography, which is less accurate than liver biopsies to determine liver fibrosis. PBMCs were isolated as described previously. The characteristics of study participants are summarized in Table 1. In accordance with METAVIR stage, individuals with advanced fibrosis presented higher indicators of liver stiffness, AST-platelet ratio index (APRI), AST, and total bilirubin than those with mild fibrosis, as well as a lower platelet count.

**Table 1.** Sample Characteristics at Baseline

	Median (IQR) or No. (%)		
Characteristic	F0/F1 (n = 27)	F4 (n = 27)	*P* Value^a^
Age, y	48.0 (47.5–52.0)	48 (43.8–53.2)	.766
Male sex	12 (44.4)	20 (74.1)	.027
Count, cells/μL			
CD4	698 (473–900)	596 (352–789)	.341
CD8	903 (618–1253)	912 (659–1438)	.850
NK cells, %	8.57 (4.92–14.08)	6.09 (2.97–14.30)	.171
CD4/CD8 ratio	0.70 (0.55–1.05)	0.61 (0.32–1.03)	.410
Time of infection, y			
HCV	14 (8–22)	17 (12–22)	.339
HIV	20 (13–23)	21 (19–22)	.685
HCV viral load, log_10_ copies	6.30 (5.93–6.68)	6.28 (5.94–6.80)	.941
Time on ART, y	13 (9–17)	13 (9–18)	.919
Routes of transmission			…
Injecting drug user	12 (44.4)	12 (44.4)	
Heterosexual	2 (7.4)	5 (18.5)	
MSM	…	1 (3.7)	
Mother to child	1 (3.7)	…	
Unknown	12 (44.4)	9 (33.3)	
HCV genotype			…
1a	17 (63.0)	13 (48.1)	
1b	3 (11.1)	2 (7.4)	
1	2 (7.4)	1 (3.7)	
2	…	1 (3.7)	
3	…	3 (11.1)	
4	…	1 (3.7)	
Unknown	5 (18.5)	6 (22.2)	
Liver stiffness, kPa	5.80 (4.95–7.05)	20.6 (15.0–25.4)	<.0001
APRI score	0.45 (0.31–0.86)	1.59 (0.56–2.11)	.0009
ALT, IU/L	65.0 (47.0–85.5)	79.5 (49.5–114.5)	.291
AST, IU/L	41.0 (33.5–74.0)	79.0 (46.8–115.8)	.009
Albumin, g/dL	4.3 (4.0–4.5)	4.3 (3.7–4.5)	.623
Platelets, x10^3^/mm^3^	188 (172–239)	144 (106–198)	.014
Total bilirubin, μg/dL	0.5 (0.3–0.6)	0.9 (0.7–1.0)	.0003

Abbreviations: ALT, alanine aminotransferase; APRI, AST-platelet ratio index; ART, antiretroviral therapy; AST, aspartate transaminase; HCV, hepatitis C virus; MSM, men who have sex with men; NK, natural killer.

^a^For median (IQR), *P* value was determined by Mann-Whitney test. For No. (%), *P* value was determined by unpaired chi-square test.

### PD-1 Expression in NK Cells Is Associated With Cell Activation and Exhaustion in Individuals Coinfected With HIV/HCV

**Figure 1.**
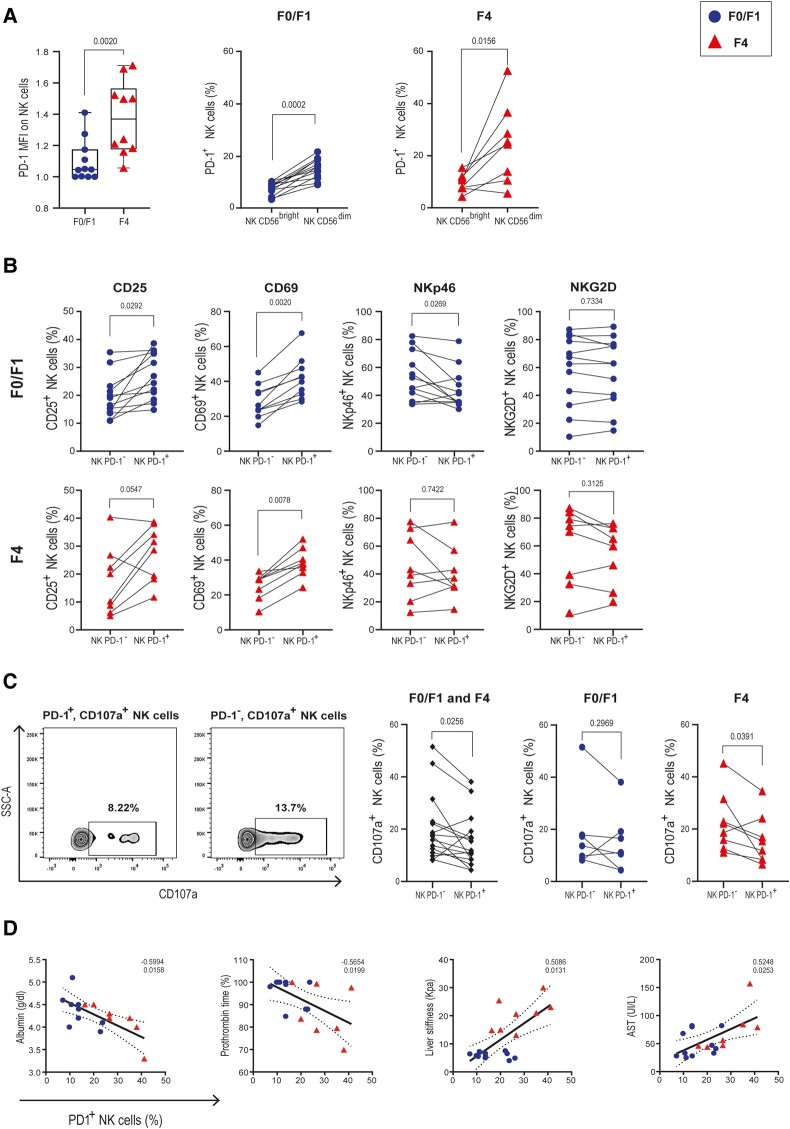
Functionality of PD-1^+^ NK cells from individuals coinfected with HIV/HCV with mild and advanced liver fibrosis at baseline, before DAA treatment. *A*, Expression of PD-1 was evaluated by flow cytometry in resting PBMCs from healthy patients and those coinfected with HIV/HCV with METAVIR F0/F1 or F4 scores. NK cells were identified as CD3^−^/CD56^+^ lymphocytes. The MFI of PD-1 expression on NK cells from the F0/F1 and F4 groups is shown with the frequency of PD-1^+^ cells in CD56^dim^ and CD56^bright^ subsets. *B*, Expression of CD25, CD69, Nkp46, NKG2D, and PD-1 was evaluated in resting PBMCs by flow cytometry. Frequency of NK cells positive for each activation marker is shown according to PD-1 status in F0/F1 (upper panel) or F4 (bottom) scores. *C*, CD107a externalization in PBMCs from participants coinfected with HIV/HCV that were incubated with K562 cells. Representative cytometry plots for CD107a expression after stimulation are shown for PD-1^−^ and PD-1^+^ NK cells (left panel). Frequency of CD107a^+^ NK cells according to PD-1 expression in the whole population (middle panel) and the F0/F1 and F4 groups (right). *D*, Correlation between frequency of PD-1^+^ NK cells and several clinicopathologic characteristics. In each graph, the correlation coefficient and *P* values are shown. Blue and red points represent mild and advanced fibrosis, respectively. Data in bar graphs are presented as median (line), IQR (box), and range (error bars). Statistical comparisons were performed with the (*A*–*C*) Wilcoxon matched-pairs signed rank test and (*D*) Spearman correlation. Each set of points represents a different person. DAA, direct-acting antiviral; HCV, hepatitis C virus; MFI, median fluorescence intensity; NK, natural killer; PBMC, peripheral blood mononuclear cell.

In comparison with individuals coinfected with HIV/HCV with mild fibrosis, NK cells from those with advanced fibrosis displayed a higher median fluorescence intensity of PD-1 (Figure 1*A*). Also, the frequency of NK/PD-1^+^ cells peaked in advanced liver fibrosis as previously demonstrated [15]. When PD-1 expression was further analyzed in peripheral NK cells from participants coinfected with HIV/HCV, PD-1 mainly subscribed to the CD56^dim^ NK cell subset, as described by others [24–26]. Evaluation of the activation markers CD25 and CD69 showed that PD-1 expression was significantly associated with an activated NK cell phenotype in persons with mild and advanced hepatic fibrosis. In addition to this result, a reduction in Nkp46 expression was registered in NK/PD-1^+^ cells from those with mild fibrosis. No differences were found in NKG2D expression between NK/PD-1^+^ and NK/PD-1^−^ cells from cases of mild or advanced fibrosis (Figure 1*B*). To evaluate whether PD-1 expression was linked to impaired NK cell functionality, degranulation capacity (externalization of CD107a) of NK/PD-1^+^ and NK/PD-1^−^ cells was studied as previously described [15]. Briefly, following stimulation with K562 cells, externalization of CD107a was monitored. When compared with the PD-1^−^ NK cell subset, expression of CD107a was significantly affected in PD-1^+^ NK cells (Figure 1*C*). Stratified analysis of the F0/F1 and F4 groups suggests that impaired NK cell degranulation is more frequently observed in cases of advanced liver fibrosis. In line with these latter results, when serum biochemical variables were analyzed, the frequency of peripheral NK/PD-1^+^ cells was negatively associated with albumin levels and prothrombin time and directly correlated with the liver stiffness, APRI score, and AST levels of individuals coinfected with HIV/HCV (Figure 1*D*). In sum, these results suggest that not only is PD-1 a marker for mature and activated NK cells but it is also linked to an exhausted phenotype and to more altered liver function tests.

### PD-1 Is a Selective Marker of NK Cell Exhaustion in Individuals Coinfected With HIV/HCV With Liver Fibrosis

**Figure 2.**
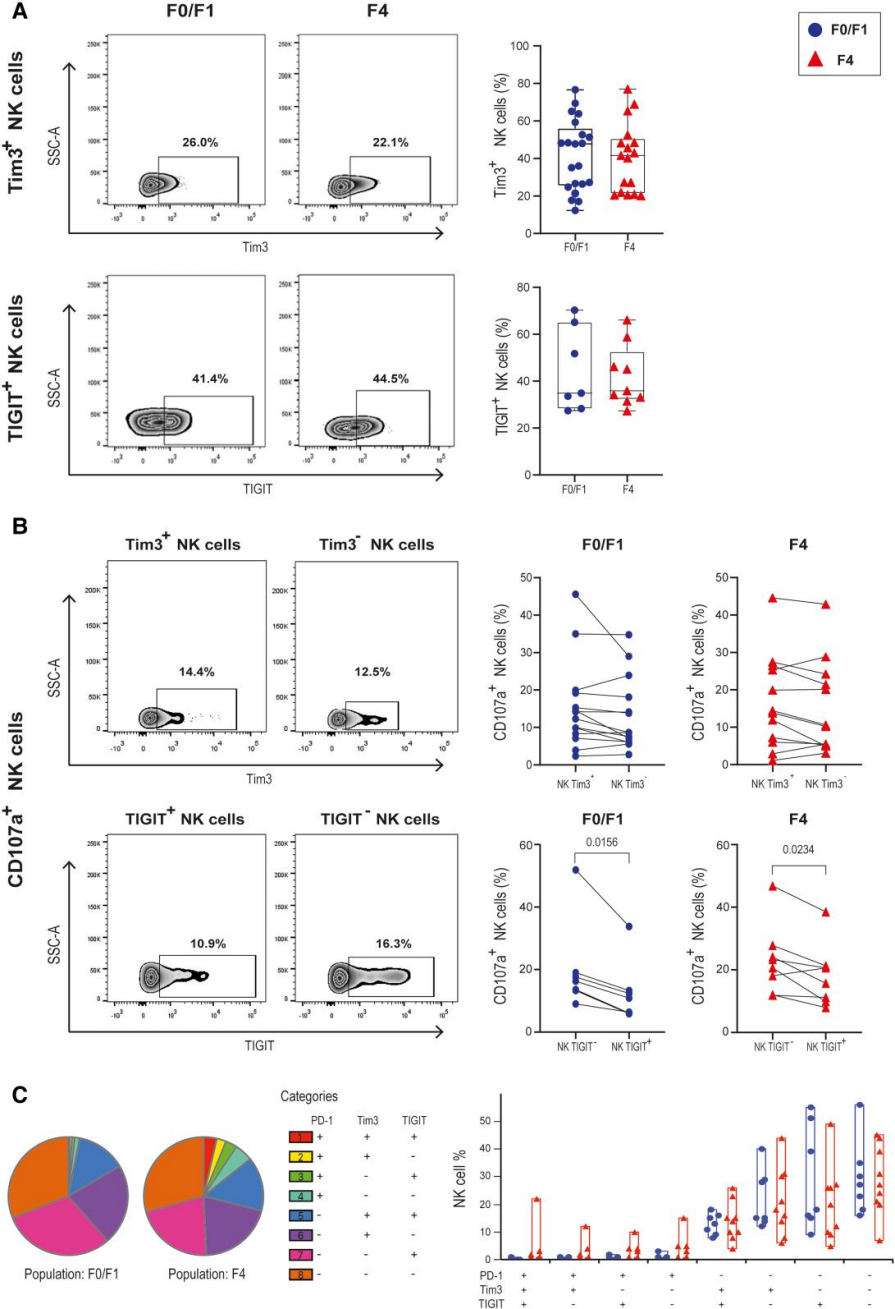
Coexpression of PD-1, Tim3, and TIGIT in NK cells from individuals coinfected with HIV/HCV and mild or advanced liver fibrosis. *A*, Expression of Tim3 (top) and TIGIT (bottom) was evaluated by flow cytometry in resting PBMCs from participants with HIV/HCV coinfection and METAVIR F0/F1 or F4 scores. Representative cytometry plots are shown on the left. Individual NK cell frequencies are indicated with the median (line), IQR (box), and range (error bars). *B*, CD107a externalization in PBMCs from cases of HIV/HCV coinfection that were incubated with K562 cells. Representative cytometry plots for CD107a expression after stimulation are shown for NK cell populations positive and negative for Tim3 and TIGIT (left). Frequency of CD107a^+^ NK cells according to Tim3 or TIGIT expression in F0/F1 and F4 groups (right). Each set of points represents a different person. *C*, PD-1, Tim3, and TIGIT coexpression analysis in those with METAVIR F0/F1 or F4 scores, according to SPICE 6.0 software. NK cell frequencies are indicated. Statistical comparisons were performed with (*A*) the Mann-Whitney test, (*B*) the Wilcoxon matched-pairs signed rank, (*C*, left) permutation analysis, and (*C*, right) the Wilcoxon rank sum test. HCV, hepatitis C virus; NK, natural killer; PBMC, peripheral blood mononuclear cell.

Next, we evaluated if PD-1^+^–exhausted NK cells expressed additional markers of dysfunction. Tim3 and TIGIT have been reported as NK cell exhaustion markers in hepatic diseases [22, 27–29]. To characterize Tim3 and TIGIT expression in NK cells from individuals coinfected with HIV/HCV, resting PBMCs were analyzed via flow cytometry. When frequencies of Tim3^+^ or TIGIT^+^ NK cells were compared, no differences were found between groups with mild and advanced fibrosis (Figure 2*A*). Similar results were obtained when median fluorescence intensity was analyzed (results not shown). To additionally identify alterations in cell functionality due to Tim3 or TIGIT expression, a CD107a degranulation assay was performed as described previously. While similar frequencies of CD107a^+^ NK cells were registered by Tim3 expression (Figure 2*B*), in cases of mild and advanced liver fibrosis, the proportion of degranulating NK cells was significantly reduced when TIGIT was expressed. Finally, we studied the distribution of NK cell populations defined by the expression of PD-1, Tim3, and TIGIT in individuals coinfected with HIV/HCV with mild and advanced liver fibrosis. Although there was a visible expansion of NK cell populations expressing PD-1 and either of the other markers among those with advanced fibrosis, the global distribution between the F0/F1 and F4 groups did not differ statistically (Figure 2*C*). Nonetheless, the coexpression of PD-1, Tim3, and TIGIT is rarely observed in NK cells in both groups studied. In sum, results show that PD-1 and TIGIT are associated with impaired NK cell functionality in persons coinfected with HIV/HCV, but only PD-1 is differentially expressed throughout the liver fibrosis stages.

### Exhaustion and Dysfunctionality of NK Cells From Individuals Coinfected With HIV/HCV With Advanced Liver Fibrosis Are Not Restored by DAA Treatment

Liver fibrosis in individuals coinfected with HIV/HCV is associated with functional exhaustion of the NK compartment and a reduction in the frequency of such cells. To evaluate whether HCV clearance with DAA affects the exhaustion and functionality of NK cells and further explore a possible role of hepatic fibrosis on this phenomenon, the frequency, PD-1 expression, and degranulation capacity of NK cells were evaluated in those coinfected with HIV/HCV with mild and advanced fibrosis treated with DAAs. Immunophenotype was studied at baseline, EOT, and 12MPT with anti-HCV therapy. Clinical characteristics of the sample are depicted in Table 2. Briefly, at 12MPT, those with advanced fibrosis presented lower counts of CD4 cells than those with mild fibrosis, which is also reflected in a lower CD4/CD8 ratio. As expected, in case of advanced fibrosis, ALT and AST levels diminished at EOT and 12MPT. Consequently, APRI score diminished too.

**Table 2.** Sample Characteristics for the Longitudinal Analysis (n = 26)

	Median (IQR) or No. (%)						
	F0/F1 (n = 14)			F4 (n = 12)			
Characteristic	BSL	EOT	12MPT	BSL	EOT	12MPT	*P* Value^a^
Age, y	49.5 (48.0–55.0)	…	…	46 (43–52)	…	…	.117
Male sex	5 (35.71)	…	…	8 (66.67)		…	.116
Count, cells/μL							
CD4	698 (570–750)	974 (314–1549)	773 (640–1061)	384 (291–776)	623 (360–856)	334 (108–499)	.029 (F0/F1 vs F4, 12MPT)
CD8	869 (780–1041)	729 (450–1054)	924 (809–979)	946 (783–1374)	1089 (1006–1307)	965 (350–1569)	.710 (F0/F1 BSL vs 12MPT)
							.715 (F4 BSL vs 12MPT)
NK cells, %	11.75 (6.27–22.28)	11.15 (9.30–17.15)	18.0 (9.6–22.0)	5.61 (3.52–10.73)	9.07 (4.97–19.03)	17.40 (9.65–23.35)	.0393 (F4 BSL vs 12MPT)
							.069 (F0/F1 vs F4, BSL)
CD4/CD8 ratio	0.78 (0.60–1.12)	0.72 (0.50–1.21)	0.80 (0.65–1.20)	0.50 (0.31–0.94)	0.64 (0.43–0.82)	0.34 (0.23–0.55)	.006 (F0/F1 vs F4, 12MPT)
Time of infection, y							
HCV	15.0 (9.5–19.8)	…	…	14 (12–22)	…	…	.805
HIV	21.0 (14.3–25.5)	…	…	19.0 (13.5–22.0)	…	…	.453
HCV viral load, log_10_ copies	6.27 (5.98–6.80)	All <1.3	All <1.3	6.04 (5.90–6.60)	All <1.3	All <1.3	.345 (F0/F1 vs F4, BSL)
Time on ART, y	14.0 (10.3–19.3)	…	…	10.5 (6.3–17.5)	…	…	.349
Routes of transmission							
Injecting drug user	7 (50)	…	…	6 (50)	…	…	
Heterosexual	1 (7.1)	…	…	2 (16.7)	…	…	
ND	6 (42.9)	…	…	4 (33.3)	…	…	
HCV genotype							
1a	8 (57.1)	…	…	7 (58.33)	…	…	
1b	2 (14.3)	…	…	2 (16.7)	…	…	
1	2 (14.3)	…	…	1 (8.33)	…	…	
3	…	…	…	1 (8.33)	…	…	
ND	2 (14.3)	…	…	1 (8.33)	…	…	
Liver stiffness, kPa	5.5 (4.8–6.2)	ND	ND	15.7 (12.4–19.0)	ND	ND	<.0001
APRI score	0.52 (0.30–0.86)	0.36 (0.17–0.38)	0.30 (0.05–0.46)	1.70 (0.79–2.30)	0.60 (0.24–0.95)	0.50 (0.30–0.86)	.090 (F0/F1 BSL vs 12MPT)
							.031 (F0/F1 vs F4, 12MPT)
ALT, IU/L	82 (53–89)	22 (21–26)	28 (23–41)	87 (72–115)	ND	25 (18–40)	.016 (F4 BSL vs 12MPT)
							.414 (F0/F1 vs F4, BSL)
AST, IU/L	47 (33–68)	18 (15–25)	24 (22–38)	90 (70–127)	ND	33 (22–38)	.016 (F4 BSL vs 12MPT)
							.026 (F0/F1 vs F4, BSL)
Albumin, g/dL	4.30 (3.95–4.45)	ND	4.7 (4.4–4.9)	4.30 (3.60–4.53)	4.4 (4.1–4.8)	4.4 (4.1–5.0)	.313 (F0/F1 BSL vs 12MPT)
Platelets, ×10^3^/mm^3^	176 (163–208)	166 (126–219)	195 (155–235)	142 (106–236)	143 (105–245)	151 (125–193)	.219 (F0/F1 BSL vs 12MPT)
							.366 (F0/F1 vs F4, BSL)
Total bilirubin, μg/dL	0.5 (0.3–0.7)	0.3 (0.2–0.5)	0.4 (0.4–0.9)	0.85 (0.56–0.98)	0.7 (0.5–1.0)	0.70 (0.60–0.88)	.051 (F0/F1 vs F4, BSL

Abbreviations: 12MPT, 12 months posttreatment; ALT, alanine aminotransferase; APRI, AST-platelet ratio index; ART, antiretroviral therapy; AST, aspartate transaminase; BSL, baseline; EOT, end of treatment; HCV, hepatitis C virus; MSM, men who have sex with men; ND, not determined; NK, natural killer.

^a^Wilcoxon and Mann-Whitney tests were used to determine *P* values as appropriate.

**Figure 3.**
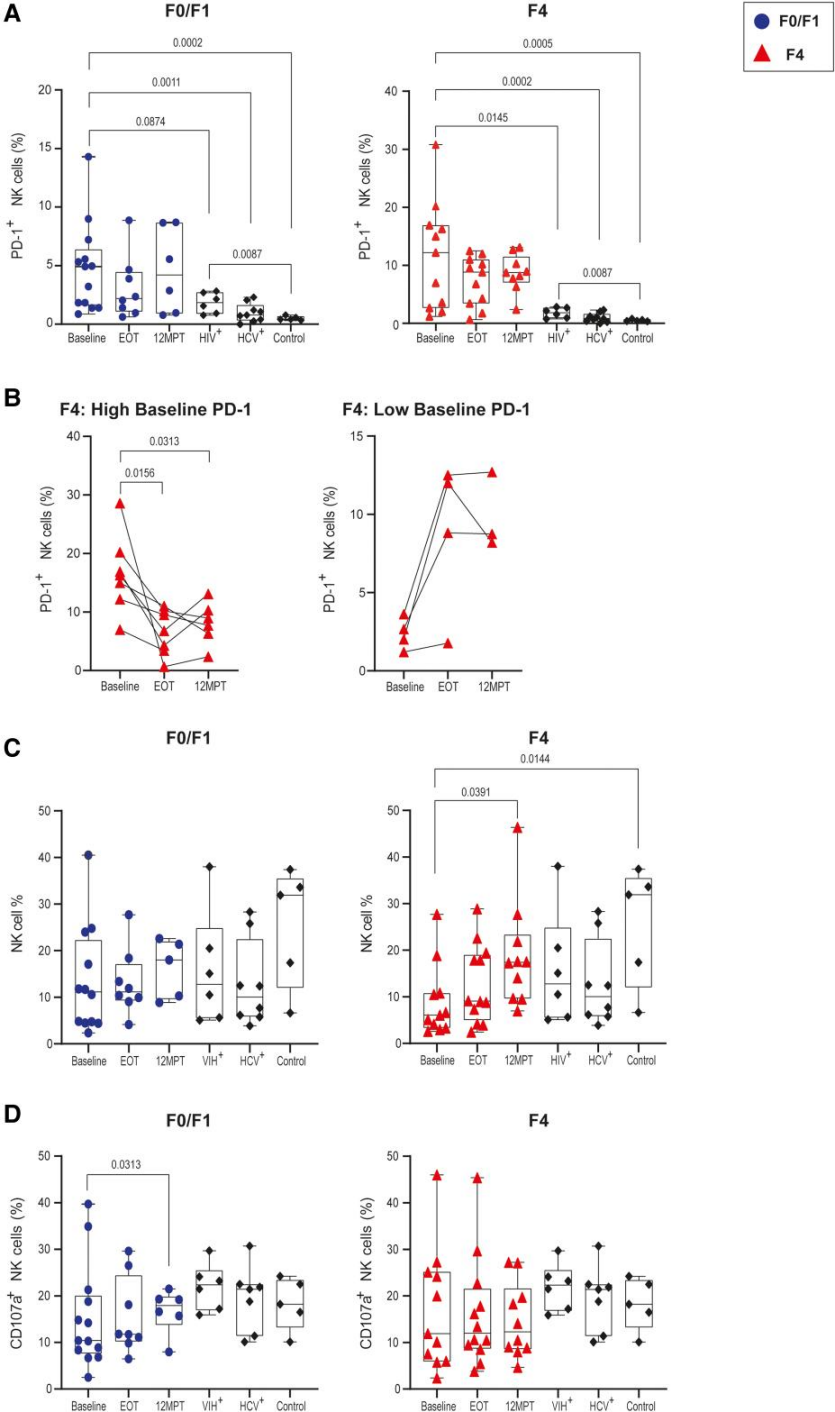
Evolution of the NK cell compartment after DAA treatment in individuals coinfected with HIV/HCV and minimal or advanced liver fibrosis. *A*, Frequency of PD-1^+^ NK cells in those with METAVIR F0/F1 or F4 scores before DAA treatment (baseline), at the end of treatment (EOT), and 12 months posttreatment (12MPT). PD-1^+^ NK cell percentage is also shown for cases of HIV (HIV^+^) and HCV (HCV^+^) monoinfection as well as healthy controls. *B*, Frequency of NK/PD-1^+^ cells in participants with METAVIR F4 scores with baseline PD-1 expression: high (>5%, left) or low (<5%, right). *C*, NK cell percentage in those with METAVIR F0/F1 or F4 scores at baseline, EOT, and 12MPT; those who were HIV^+^ or HCV^+^; and healthy donors. *D*, CD107a externalization assessed in NK cells from participants with METAVIR F0/F1 or F4 scores at baseline, EOT, and 12MPT after incubation with K562 cells. Frequency of NK/CD107a^+^ cells is also shown for HIV^+^ or HCV^+^ cases and healthy controls. Data in bar graphs are presented as median (line), IQR (box), and range (error bars). Statistical comparisons were performed with the Wilcoxon and Mann-Whitney test. DAA, direct-acting antiviral; HCV, hepatitis C virus; NK, natural killer.

First, we evaluated PD-1 expression dynamics over time in the F0/F1 and F4 groups. As seen in Figure 3*A*, baseline levels of PD-1 expression in HIV/HCV are significantly higher than those displayed in HCV or HIV monoinfection or healthy control. Although all achieved a sustained virologic response after DAA treatment, globally, exhaustion of NK cells was not modified. Interestingly, when cases of advanced fibrosis were evaluated, HCV clearance was associated with decreased cell exhaustion in individuals with NK cells showing high PD-1 expression at baseline. On the contrary, for those few who had a low frequency of PD-1^+^ cells, the expression of this marker increased over time, although not significantly (Figure 3*B*). NK cell percentage and degranulation capacity were not directly associated in either of the liver fibrosis stages evaluated. While the frequency of NK cells did not change after DAA treatment in participants with mild fibrosis, the degranulation capacity was significantly improved (Figure 3*C* and 3*D*, left panel). In cases of advanced fibrosis, although the percentage of those cells was restored, loss of functionality was not.

## DISCUSSION

The arrival of DAA treatment in the last decade has represented a great advance in HCV treatment, since viral clearance is possible in >90% of cases, with minimal adverse effects and short treatment schedules (2–3 months) [7]. However, the capacity of DAA therapy to improve liver fibrosis, especially in cases of advanced liver disease, is less clear [8, 30]. Liver fibrosis is directly correlated with increased morbidity and mortality due to liver failure and hepatocellular carcinoma [31–33]. As advanced fibrosis persists, HCV elimination is not enough to restore health, and options to decrease the level of liver damage are needed. NK cells play a crucial role in the modulation of hepatic stellate cells, key cells in the generation of liver fibrosis. In the present study, we were able to add evidence regarding NK cells and their association with liver fibrosis in individuals coinfected with HIV/HCV. We have shown that NK cells from cases of HIV/HCV coinfection with advanced hepatic fibrosis express higher levels of PD-1 than those with mild liver disease (Figure 1*A*). Also, we demonstrated that PD-1 is significantly associated with a lower capacity of NK cells to degranulate (Figure 1*C*). The possibility of increasing NK cell functionality could benefit individuals and have an impact on liver tissue remodeling. Since the improvement of NK cell degranulation capacity was observed only in samples with lower fibrosis, this underscores the importance of designing strategies that could apply as possible treatments (eg, anti-PD-1 that has already been approved as adjunctive therapy to chemotherapy) to revert exhaustion and modify in vivo the function of NK cells.

Regarding additional markers of NK cell exhaustion that have been reported, we noted that the expression of TIGIT was associated with a decrease in impaired degranulation in cases of mild and advanced fibrosis (Figure 2*B*). Nevertheless, TIGIT was not differentially expressed on NK cells from individuals with different degrees of liver fibrosis (Figure 2*A*). Anti-TIGIT therapies are being studied for cancer treatment [34], so it is plausible to consider them as a therapy to improve NK functionality and secondary liver fibrosis. Tim3 was not associated with a lower capacity of degranulation in NK cells. However, a higher percentage of NK/Tim3^+^ cells was observed in those with mild and advanced fibrosis as compared with the control group (data not shown). Future analysis should aim to completely understand the role that this marker plays in the context of monoinfection and HIV/HCV coinfection.

HCV elimination by DAA treatment may exert a differential effect on different immune parameters, depending on one’s level of liver fibrosis. Here, multiple NK cell parameters were longitudinally monitored after HCV eradication with DAA treatment by analyzing 3 sampling times: before DAA treatment (baseline), EOT, and 12MPT. In the case of mild fibrosis, an increase in NK cell degranulation capacity was noted at 12MPT (Figure 3*D*). Yet, with advanced fibrosis, this improvement in degranulation capacity was not found. Nevertheless, an increase in the frequency of NK cells was seen at 12MPT (Figure 3*C*), and a decrease in the percentage of NK/PD-1^+^ cells at EOT and 12MPT was detected (Figure 3*B*). This incomplete recovery of the NK cell compartment may suggest a persistent exhaustion of NK cells even after HCV has been eliminated by DAA treatment, particularly in those with advanced fibrosis, who are unable to improve their NK cell degranulation capacity. This is consistent with data reported by other groups describing a limited capacity of DAA treatments to revert the METAVIR score in those with F3 and F4 [35–37]. These data reinforce the possibility to use the aforementioned immune checkpoint inhibitors to improve the functionality of NK cells and, as a consequence, modify their impact on liver tissue fibrosis.

When modulation of PD-1 expression in NK cells was evaluated following DAA therapy, no significant overall changes were documented in samples with mild or advanced fibrosis. Nevertheless, while NK/PD-1^+^ cell frequency is significantly higher at baseline than in control groups, this significance is lost after DAA treatment, suggesting a trend that could be better elucidated with a larger sample size and at longer follow-ups. Additionally, when analyzing samples with advanced fibrosis, we observed that PD-1 expression at baseline was heterogenous, and we also observed different outcomes over time. In cases of F4 with high baseline expression of PD-1, a decrease of NK/PD-1^+^ cell frequency was noted at EOT and 12MPT vs baseline (Figure 3*B*, left panel). This result was as expected, as it has been found in the cohorts with advanced fibrosis [12, 15] and could indicate an improvement in NK cell homeostasis; however, this does not seem to be enough to restore its NK cell degranulation capacity since no significant changes in this parameter were detected over time. With respect to individuals with a low baseline percentage of NK/PD-1^+^ cells, a paradoxical behavior is noted, since this frequency increases after HCV clearance (Figure 3*B*). In these participants with low PD-1 baseline expression in their NK cells, high cellular death was documented in the viability control in the flow cytometry analysis. Therefore, one possible explanation for this observation is that in those samples, NK cells presented a high level of exhaustion before DAA and died preferentially during the in vitro experimental conditions. If this is the case, the real proportion of PD-1^+^ NK cells would be underestimated in these samples and could explain the paradoxical outcome seen in this group. As for those with mild fibrosis, although there was no significant decrease in the percentage of NK PD-1^+^ cells as a function of time (Figure 3*A*), there was a significant increase in the degranulation capacity of NK cells at 12MPT (Figure 3*D*). This would suggest that at least in people with mild fibrosis, it is possible to recover the functionality of NK cells once HCV is cleared with DAA.

From the results reported in the present article, it could be inferred that the presence of advanced liver damage exerts a direct effect on NK cells, regardless of the presence of HCV. From our results, PD-1 and TIGIT emerged as markers of exhaustion in NK cells and compromised degranulation capacity. However, only PD-1 was differentially expressed, with a higher percentage of PD-1^+^ cells in individuals with advanced fibrosis. This observation could reflect more pronounced immune system exhaustion in people with advanced fibrosis. In line with this, it has been reported that those NK cells that coexpress Tim3 and TIGIT are increased in persons with advanced fibrosis [28].

One of the limitations that faced this study included the great heterogeneity of the data obtained from samples of HIV/HCV coinfection with mild and advanced fibrosis. A larger sample size could help to elucidate the role of each of the studied markers more clearly. Another limitation is that the functionality of NK cells was evaluated ex vivo, under conditions that did not allow taking into account the multiple interactions that occur within the organism, at the level of direct cellular interactions and soluble molecules, such as cytokines.

## CONCLUSIONS

The results suggest that DAA therapy is not sufficient per se to reverse the state of general exhaustion of the NK cells and their loss of functionality in the short term, particularly in people with advanced fibrosis. Nevertheless, they shed light on possible modifiable immune parameters that could be involved in NK cell alterations. These results highlight the importance of the active search of new therapies (eg, checkpoint inhibitors) that allow reversion of the described exhausted immune phenotype, which may improve the level of liver fibrosis and reduce the risk of mortality associated with it.

## Supplementary Data

Supplementary materials are available at *Open Forum Infectious Diseases* online. Consisting of data provided by the authors to benefit the reader, the posted materials are not copyedited and are the sole responsibility of the authors, so questions or comments should be addressed to the corresponding author.

## Supplementary Material

ofad591_Supplementary_Data

## Notes

***Acknowledgments***. We gratefully thank all participants of the cohorts who donate their samples for this study.

***Author contributions***. A. O., M. L. P., and N. L. contributed to study conceptualization, investigation, formal analysis, and writing original draft. A. O., M. L. P., L. B., A. U., and N. L. contributed to data acquisition. A. O., M. L. P., L. B., A. U., Y. G., V. G. P., G. T., M. F. Q., and N. L. contributed to data analysis and methodology. G. P. and S. P. contributed to participants’ enrollment and clinical data analysis. N. L. conducted the study and was in charge of supervision, funding acquisition, project administration, monitoring of enrolled participants, and collecting of clinical data. All authors contributed to and approved of the final version of the manuscript.

***Disclaimer.*** The funders had no role in the study design, data collection and interpretation, or the decision to submit the manuscript for publication.

***Ethics*.** The study was conducted in accordance with the Declaration of Helsinki and was reviewed and approved by the Bioethics Committee of Fundación Huésped.

***Patient consent statement*.** Individuals were invited to participate at Instituto de Investigaciones Biomédicas en Retrovirus y SIDA. Study participants provided written informed consent prior to enrollment and for use of their data and samples for future studies.

***Financial support*.** This work was supported by the Agencia Nacional de Promoción Científica y Tecnológica (Grant PICT-2019-0042).

***Potential conflicts of interest*.** All authors: No reported conflicts.
