# Supplementary material for: Markers of Natural Killer Cell Exhaustion in HIV/HCV Coinfection and Their Dynamics After HCV Clearance Mediated by Direct-Acting Antivirals
Source: Open Forum Infect Dis. 2023 Nov 22;10(12):ofad591. doi: 10.1093/ofid/ofad591 (PMC10723816; doi:10.1093/ofid/ofad591)
Supplement: ofad591_Supplementary_Data [file ofad591_supplementary_data.zip › Supplemental material Osegueda et al..docx]

# SUPPLEMENTARY FIGURES AND TABLES

**Supplementary Table 1. Fluorochrome-conjugated antibody panels.**

| **Antibody** | **Fluorochrome** | **Clone** | **Supplier** |
| --- | --- | --- | --- |
| **NK functional assays** | | | |
| Anti-CD107a | FITC | H4A3 | Biolegend |
| Anti-CD3 | PE-CY7 | SK7 | Biolegend |
| Anti-CD56 | PE-CY5 | 679.1Mc7 | Beckman Coulter |
| **NK Immunophenotyping** | | | |
| Anti-CD3 | BV605 | SK7 | BD Biosciences |
| Anti-PD-1 | BV421 | EH12.1 | BD Biosciences |
| Anti-TIGIT | PE-CY7 | A15153G | Biolegend |
| Anti-Tim3 | FITC | F38-2E2 | Biolegend |
| Anti-CD25 | PE | BC96 | Biolegend |
| Anti-CD69 | FITC | FN50 | Biolegend |
| Anti-Nkp46 | PE-CY7 | 9E2 | Biolegend |
| Anti-NKG2D | APC | 1D11 | Biolegend |

**Supplementary Figure 1. Gating strategy and representative dot plots for flow cytometry analysis. A:** Upon incubation with cRPMI, immunophenotype, functionality and exhaustion markers were studied. NK cell subset was defined as CD3-/CD56+ viable lymphocytes. At least 3000 events were acquired for NK-cell gate for the subsequent analysis. Degranulation activity of NK-cells was determined as NK/CD107a+ subset. **B:** PD-1, Tim3 and TIGIT expression was studied on NK-cell population after incubation with cRPMI. **C:** Upon coincubation with K562 cells, CD107a expression was determined in NK-cells expressing or not PD-1, Tim3 and TIGIT. Dot plots from one representative cytometry are shown.
