# Supplementary figures and images for: Markers of Natural Killer Cell Exhaustion in HIV/HCV Coinfection and Their Dynamics After HCV Clearance Mediated by Direct-Acting Antivirals
Source: Open Forum Infect Dis. 2023 Nov 22;10(12):ofad591. doi: 10.1093/ofid/ofad591 (PMC10723816; doi:10.1093/ofid/ofad591)

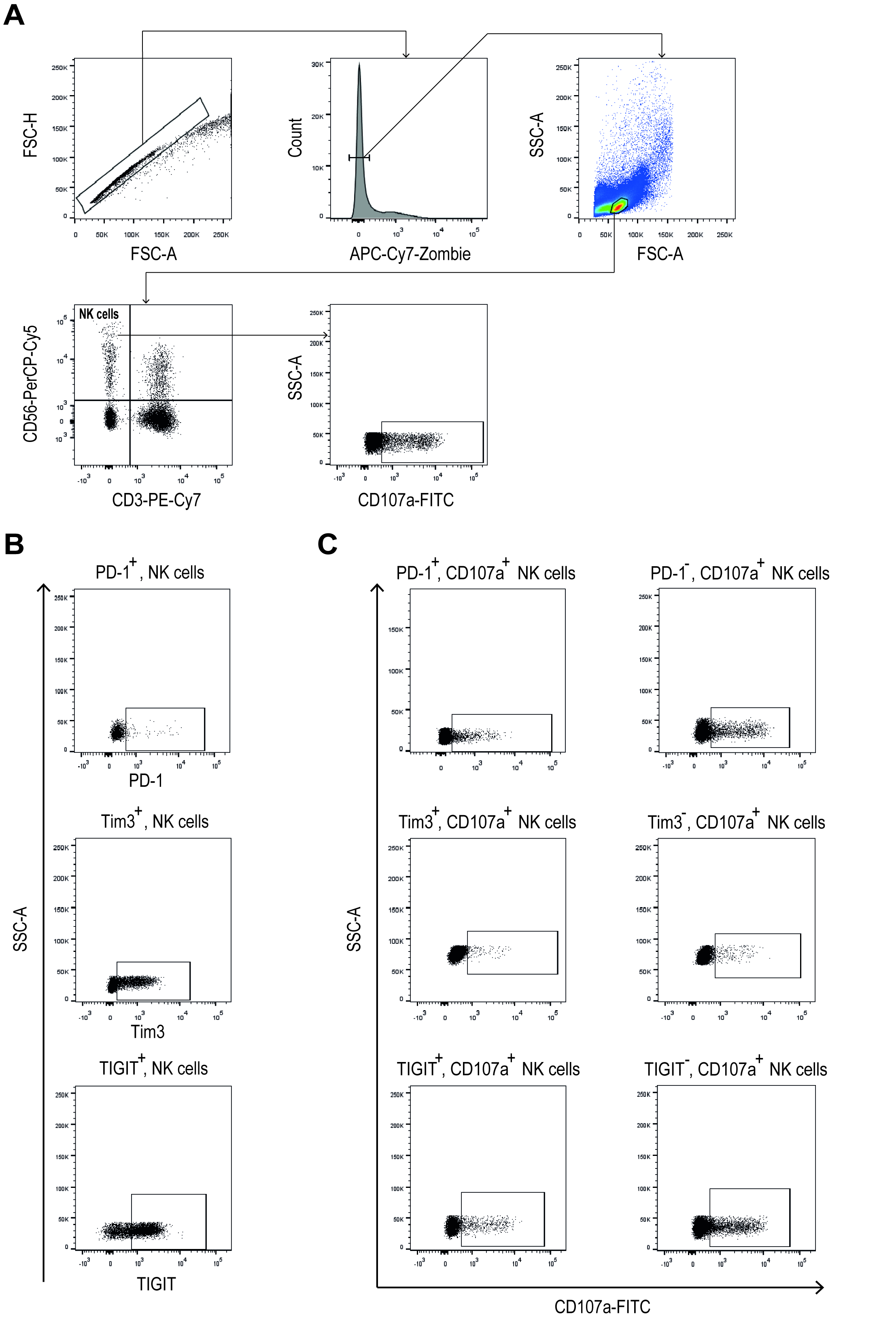

Supplement: ofad591_Supplementary_Data [file ofad591_supplementary_data.zip › Supplementary Figure 1 Osegueda et al..tif]
